# Patchy ‘coherence’: using normalization process theory to evaluate a multi-faceted shared decision making implementation program (MAGIC)

**DOI:** 10.1186/1748-5908-8-102

**Published:** 2013-09-05

**Authors:** Amy Lloyd, Natalie Joseph-Williams, Adrian Edwards, Andrew Rix, Glyn Elwyn

**Affiliations:** 1 Institute of Primary Care and Public Health, Cardiff University School of Medicine, Heath Park, Cardiff, CF, 14 4YS, UK; 2 The Dartmouth Center for Health Care Delivery Science, HB 7256, 37 Dewey Field Road, 2nd Floor, Hanover, NH 03755, USA; 3 Independent advisor to the MAGIC program Institute of Primary Care and Public Health, Cardiff University School of Medicine, Health Park, Cardiff, CF, 14 4YS, UK

**Keywords:** Shared decision making, Implementation, Patient-centered care, Normalization Process Theory

## Abstract

**Background:**

Implementing shared decision making into routine practice is proving difficult, despite considerable interest from policy-makers, and is far more complex than merely making decision support interventions available to patients. Few have reported successful implementation beyond research studies. MAking Good Decisions In Collaboration (MAGIC) is a multi-faceted implementation program, commissioned by The Health Foundation (UK), to examine how best to put shared decision making into routine practice. In this paper, we investigate healthcare professionals’ perspectives on implementing shared decision making during the MAGIC program, to examine the work required to implement shared decision making and to inform future efforts.

**Methods:**

The MAGIC program approached implementation of shared decision making by initiating a range of interventions including: providing workshops; facilitating development of brief decision support tools (Option Grids); initiating a patient activation campaign (‘Ask 3 Questions’); gathering feedback using Decision Quality Measures; providing clinical leads meetings, learning events, and feedback sessions; and obtaining executive board level support. At 9 and 15 months (May and November 2011), two rounds of semi-structured interviews were conducted with healthcare professionals in three secondary care teams to explore views on the impact of these interventions. Interview data were coded by two reviewers using a framework derived from the Normalization Process Theory.

**Results:**

A total of 54 interviews were completed with 31 healthcare professionals. Partial implementation of shared decision making could be explained using the four components of the Normalization Process Theory: ‘coherence,’ ‘cognitive participation,’ ‘collective action,’ and ‘reflexive monitoring.’ Shared decision making was integrated into routine practice when clinical teams shared coherent views of role and purpose (‘coherence’). Shared decision making was facilitated when teams engaged in developing and delivering interventions (‘cognitive participation’), and when those interventions fit with existing skill sets and organizational priorities (‘collective action’) resulting in demonstrable improvements to practice (‘reflexive monitoring’). The implementation process uncovered diverse and conflicting attitudes toward shared decision making; ‘coherence’ was often missing.

**Conclusions:**

The study showed that implementation of shared decision making is more complex than the delivery of patient decision support interventions to patients, a portrayal that often goes unquestioned. Normalizing shared decision making requires intensive work to ensure teams have a shared understanding of the purpose of involving patients in decisions, and undergo the attitudinal shifts that many health professionals feel are required when comprehension goes beyond initial interpretations. Divergent views on the value of engaging patients in decisions remain a significant barrier to implementation.

## Background

Implementing shared decision making (SDM) in routine practice is difficult [1], despite considerable interest from policy-makers. In SDM, clinicians and patients are expected to make decisions together, using the best available evidence. Patients are encouraged to think about the available screening, treatment, or management options, and the likely benefits and harms of each so that they can communicate their preferences and collaborate to select the best course of action [2]. Most attempts by researchers to implement SDM have been based on a narrow interpretation that SDM [3] is the delivery of decision support tools to patients in hopes of accomplishing greater levels of patient-provider collaboration. Over 80 randomized controlled trials, mostly conducted since the early 1990s onwards, have demonstrated that decision support tools lead to patients having greater knowledge, more accurate risk perceptions, and greater comfort with decisions, while also reducing the number of patients remaining undecided or choosing major surgery [4]. Yet, debate remains as to whether greater levels of collaboration are generated within clinical encounters. In addition, there are very few demonstrations of successful implementation of decision support tools outside of a research setting [5,6]. This ‘tool delivery’ interpretation of SDM is being challenged [3], and is viewed as too myopic.

Several key authors have provided insight into the barriers of using decision support tools [7-9]. However, there have been no reports of broader approaches to implementation. A systematic review of implementation attempts revealed conceptualizations that define SDM as the routine use of decision support tools [6], work confirmed at a recent conference [1]. We were commissioned by The Health Foundation (UK) to undertake a broader approach, which included efforts to explain the tenets of SDM to clinical teams, engage them in the co-development of tools, and enhance their skills at involving patients in SDM. This paper describes a qualitative study of health professionals’ experiences as we worked with them to attempt to embed SDM into routine care. We chose the Normalization Process Theory (NPT) as our lens, having previously used this approach to explore implementation challenges [10]. NPT is an explanatory model that helps to review and evaluate implementation processes. Our aim in this article is to use NPT to examine the work that needs to be done by health professionals to inform future efforts to implement and embed SDM.

## Methods

### The MAGIC program

This study was part of Phase I of the MAking Good Decisions In Collaboration (MAGIC) implementation program, commissioned by The Health Foundation, which involved joint work between Cardiff University School of Medicine, Cardiff and Vale University Health Board, Newcastle University, and Newcastle-upon-Tyne Hospitals NHS Foundation Trust. We worked with a variety of clinical teams across sites and settings to develop and test interventions designed to promote SDM (see Table 1). The implementation approach drew on a well-known ‘model for improvement’ [11]. In this model, core teams are asked to agree on clear aims and measures using iterative Plan-Do-Study-Act (PDSA) cycles. This study reports on healthcare professionals’ experiences of the interventions in three secondary care specialties: head and neck cancer, breast cancer, and pediatric tonsillectomy.

**Table 1:** Interventions and influences utilized during Phase 1 of MAGIC program

**Intervention**	**Description**
Team feedback tool	A 22-item questionnaire designed to elicit team members’ views of their own and their team’s levels of competence in SDM.
Introductory workshop	A one-hour overview of SDM, including theories/definitions, rationale, evidence base, and methods for implementation.
SDM questionnaire	An eight-item Likert scale questionnaire designed to obtain patients’ perceptions of the degree of their involvement in decisions.
Extended training workshop	Two-hour training session on ‘how to do’ SDM, using simulated consultation scenarios.
Option Grids	Brief within-encounter patient decision support tools designed to help compare reasonable important options [12]. In some cases Option Grids were already available [13]. The Ear, Nose and Throat teams were invited to develop their own Option Grids.
Decision Quality Measures	A 15-item questionnaire (adapted from Sepucha and colleagues [14]): assessing patients’ understanding of the key features, risks and benefits of treatment options; their readiness to decide; and their preferred choice of treatment. Some teams asked patients to complete DQMs at two time points in the decision making process to assess the impact of their new practice.
Clinical leads meetings	Monthly meetings with implementation team and clinical team leader to check progress.
Learning events	Six monthly meetings where clinical leads from primary and secondary care teams in Newcastle and Cardiff met to address implementation challenges.
Feedback sessions	Six monthly seminars with each clinical team to present data.
‘Ask 3 Questions’ campaign	Posters, leaflets and business cards designed to raise awareness of SDM and encourage both patients and doctors to work together in deciding on the best course of action (campaign adapted from the ‘Ask 3 Questions’ study in Australia [15] and accompanied by Executive Board-wide communication strategy).
Executive Board level support	Eliciting demonstrable support from the Executive Board (or similar level in primary care) and middle-management for the MAGIC Program, *e*.*g*., policies, reports, senior management interest.

### Participants and data collection

Semi-structured interviews were conducted with healthcare professionals from the three secondary care teams. All participants had direct contact with patients. The MAGIC program was considered by the Cardiff and Vale University Health Board Research Governance Committee to be an implementation program, and the data collected to be part of the evaluation of service development. As such, the program was not considered to require formal ethical approval.

Two rounds of interviews were conducted after 9 and 15 months of the 18-month program. After consenting, participants were interviewed by an experienced qualitative researcher (AL). All interviews were audio-recorded and transcribed. During the first round of interviews, participants were asked to describe their involvement with the program, and to share their opinion about the most significant change that had occurred during the first nine months. They were asked why they felt the change was significant, and how, if at all, the program had contributed. During the second round of interviews, participants were asked about the most significant developments since the beginning of the program; they were asked to share their views about SDM and to describe the extent to which they were adopting the approach, and if not, why not. They were also asked whether any systems had been set up to support or sustain SDM, and which of the program interventions had contributed the most to any change. The interview schedule drew on the most significant change (MSC) technique, an approach that has been used to monitor and evaluate complex participatory implementation programs [16].

### Data analysis: using a framework approach

Each transcript was coded independently by two researchers (AL & AR), using a framework analysis approach to assess the effort to implement SDM using the core constructs and components of NPT [17,18]. NPT provides an appropriate frame to examine the dynamics of implementing and integrating complex interventions, such as SDM, into workplace teams and has been used in many similar studies to date [19-21]. NPT proposes that ‘complex interventions become routinely embedded (implemented and integrated) in their organizational and professional contexts as the result of people working, individually and collectively, to implement them’ [22]. According to the theory, the work of implementing a complex intervention is operationalized by four generative mechanisms (see Table 2) and requires collective and continuous investment in sense-making, commitment, effort and appraisal. Once a complex intervention is routinely embedded in practice, it becomes ‘normalized.’ This theory provided a good match with our wish to examine the implementation efforts and to go beyond a barriers and facilitators approach.

**Table 2:** Four generative mechanisms of Normalization Process Theory

**Generative mechanism**	**Description**	**Components**
**Coherence**	The **sense**-**making work** that people do individually and collectively when they are faced with the problem of operationalizing some set of practices.	Differentiation
		Communal Specification
		Individual Specification
		Internalization
**Cognitive participation**	The **relational work** that people do to build and sustain a community of practice around a new technology or complex intervention.	Initiation
		Enrolment
		Legitimation
		Activation
**Collective action**	The **operational work** that people do to enact a set of practices, whether these represent a new technology or complex healthcare interventions.	Interactional Workability
		Relational Integration
		Skillset Workability
		Contextual Integration
**Reflexive monitoring**	The **appraisal work** that people do to assess and understand the ways that a new set of practices affect them and others around them.	Systemization
		Communal Appraisal
		Individual Appraisal
		Reconfiguration

Descriptive codes were generated and organized through thematic analysis under a framework based on the components of the four NPT constructs. Themes were identified using this framework and compared across the data. For example, under the construct ‘coherence,’ we coded data in relation to the components ‘differentiation’ (Is SDM different from what they already do?), ‘communal specification’ (Does the team agree on the purpose and value of SDM?), ‘individual specification’ (Does each team member understand how it affects their day-to-day roles and responsibilities?), and ‘internalization’ (Do those tasks and responsibilities make sense?).

We also examined the data for deviance to avoid overlooking issues that did not map onto the NPT framework. Inter-coder agreement was sought for all coding and the generation of categories and themes. To mitigate potential bias, both researchers (AL, AR, NJW, GE), and the leads from each of the clinical teams reviewed the transcripts.

## Results and discussion

A total of 35 front-line healthcare professionals were invited to participate in this study. Eligible participants included consultants (n = 15), nurses (n = 11), registrars (residents) (n = 3), allied health professionals (n = 3), and a consultant nurse (n = 1). In total, 54 interviews were conducted, over two rounds, with 31 front-line healthcare professionals (89% of healthcare professionals were interviewed at least once). Interviews were conducted with a range of team members, including 13 consultants (42%), 11 nurses (36%), 3 allied health professionals (10%), 3 registrars (residents) (10%), and 1 consultant nurse (2%). A total of 23 healthcare professionals were interviewed twice. In total, 8 healthcare professionals were interviewed once: 1 was unavailable at 9 months, and by 15 months, 3 had left the teams and 4 were unavailable for interview. Interview duration was between 15 to 60 minutes. All interviews were included for analysis.

The interview data highlighted the extent of cognitive and behavioral work that clinicians, managers, and policy-makers need to do, individually and collectively, to normalize SDM. No themes were identified that could not be coded using the NPT framework, and agreement was reached on all coding. Here, we identify and describe four key themes, shaped by the NPT framework (see Table 3).

**Table 3:** Themes: the work of implementation

**Descriptive themes**	**Normalization process theory generative mechanisms**
Uncovering divergent views: the challenge of building coherence	Coherence
Facilitating participation, transferring ownership of the work	Cognitive participation
Assessing fit and adapting to change together	Collective action
Monitoring benefit, reflecting on value	Reflexive monitoring

### Uncovering divergent views: the challenge of building coherence

The first construct of NPT is ‘coherence’; the sense-making work required for successful implementation. The key question to ask is, ‘what is the work?’ ‘Coherence’ was one of the key themes to emerge from the data, highlighting the importance of a clinical team sharing the same understanding about the principles of SDM, how SDM differed from their existing approach, and whether they wanted to adopt SDM in their work routines. It was clear from the interviews at nine months that the teams were usually far from agreement on most of these issues.

Members of the same team often offered conflicting views on their approach to decision making with patients. Some proposed that the team had ‘always involved patients in decision making’ (Allied Health Professional, Head and Neck Cancer); others stated that this approach was neither beneficial nor appropriate. One consultant suggested that the role of the clinical team was to protect patients from the ‘agony’ of choice: ‘I think the function of the MDT [multidisciplinary team] should definitely be to quite clearly help people to make what we think is the best decision’ (Consultant, Head and Neck Cancer).

Healthcare professionals became more aware of the lack of a coherent approach to SDM during the course of the program. During the first few months, variable understanding of the aims, objectives and expected benefits of SDM among team members was mostly unnoticed, or at least, was not of any importance. Data collected during the second round of interviews showed that MAGIC had differentiated SDM from current practice, and as a result had increased awareness that different, often strong, views prevailed: ‘What [involvement in the program] has caused lots of people to do is actually think about the process of imparting information from themselves to a patient when that patient has a choice to make…And it’s been a surprise for me how I’d always believed that we all roughly thought the same way because we’re all roughly taught the same way, but in fact we have polarized views about how that should happen’ (Consultant, Head and Neck Cancer). Some healthcare professionals rejected the principles of SDM. Others reported a change in their view of SDM as ‘something they already did,’ to ‘something that they could do better:’ ‘Initially when we started, like many of us, I thought ‘we do that anyway’. I think the biggest difference is, well actually, we didn’t do it well’ (Nurse, Breast Cancer).

Clinicians said that the most useful interventions were the short and extended training workshops; the experience of developing and implementing tangible tools, such as brief decision support tools (Option Grids); and feedback from short patient-reported questionnaires (Decision Quality Measures).

In short, the workshops differentiated SDM from the seemingly obvious (and accepted) concept of ‘involving patients.’ The ‘coherence’ was sometimes enhanced when practical tools such as Option Grids and Decision Quality Measures (DQMs) were introduced: ‘What’s different is that we’re making it more concrete now, rather than an ideal…it’s a practical thing now…we can actually put it into practice rather than just say it’s a good idea. We can encourage and show people how they can do it better and we can measure that we are doing it’ (Clinical Nurse Specialist, Breast Cancer).

However, the new awareness had a price. Some team members did not agree that treatment options should be made explicit or that patients should be asked to consider relevant trade-offs. Identifying these differing views usually led to disagreements where previously colleagues had assumed shared values. While we identified and worked with healthcare professionals who advocated for SDM, we do not think that ‘coherence’ about SDM at the level of each team was accomplished during the course of the program. This lack of coherence was an obstacle to implementation, or as the theory would propose, normalization.

### Facilitating participation, transferring ownership of the work

The second construct of NPT is called ‘cognitive participation.’ This term describes the work done by individuals in teams as they develop roles and take on tasks in accomplishing new ways of doing things. The key question to ask is, ‘Who does the work?’

The data we collected identified participation in the new set of practices as integral to successful implementation. Participants described the new SDM work as requiring leaders to define the work, and then enrolling others to contribute collectively to the process. Identifying leadership support for SDM was challenging: clinical teams are not simple hierarchical units, and substantial autonomy exists, especially for experienced clinicians. As we described above, many team members had conflicting views about the goal and value of SDM. Individuals who advocated for SDM, motivated by the prospect of improving practice, faced resistance. One clinical lead described her efforts as ‘banging [her] head against a brick wall’ (Clinical Nurse Specialist, Head and Neck).

Mediating conflicting views among team members delayed the development of interventions. Coupled with decreased support from some team members, the loss of momentum had significant impact. A consultant, reflecting on a perceived lack of progress at the second round of interviews explained that a key reason was ‘lack of time and leadership…there’s no one driving the project…it needs someone on the inside, it needs a clinician to drive it, and no one has taken that on’ (Consultant, Head and Neck).

The interview data demonstrated that the most effective way of enrolling team members was to engage them in the process of developing SDM interventions. The clinical leads, when engaged in the work, involved other team members in the development of Option Grids and DQMs. This involvement helped them reflect on their existing practice: ‘…thinking through the questions…helped us think about what we discuss at our home visits and how we do that?’ (Nurse, Breast Cancer). Others felt that the MAGIC team’s approach of working collaboratively with the clinical teams helped to legitimize the interventions, which in turn increased likelihood of implementation. As one consultant noted, being ‘open to ideas and wanting us to give our feedback’ led to the development of tools ‘that can be used on a practical level’ (Consultant, Breast Cancer). In contrast, delivering interventions without involving the clinical teams in their development was counter-productive; these were more likely to be described as ‘a complete waste of time’ (Nurse, Breast Cancer).

Working collaboratively with clinical teams helped the clinical leads to define and initiate new practices. DQMs and Option Grids were, in due course, routinely used by nurses in the breast cancer clinic and in the clinic where referrals for tonsillectomy were assessed. It was not possible to introduce these tools into the multidisciplinary team for head and neck cancer. Team members had divergent views on the role and purpose of SDM. We observed that ‘cognitive participation’ in the development of tools and measures as well as the iterative process of trying them out, and resolving problems, were fundamental to accomplishing ownership and engagement. These steps were possible but required regular external engagement, facilitation and encouragement over many months.

### Assessing fit and adapting to change together

The NPT construct ‘collective action’ can be examined by focusing on how new interventions are implemented into practice, in short, ‘how does the work get done?’ The interview data revealed that the teams were most able to grasp SDM when given tangible interventions that could be integrated into routine practice. NPT uses the terms ‘interactional workability’ to describe things that can be fitted into existing work and ‘relational integration’ to describe work done to build accountability and maintain confidence in the new set of practices. The term ‘skillset workability’ relates to the clear allocation of roles and responsibilities, and ‘contextual integration’ refers to the relationship between the interventions and established policies and procedures.

Many healthcare professionals referred (effectively) to the ‘interactional workability’ of Option Grids. Option Grids were described as tools that facilitated interaction with patients, as providing ‘structure’ for clinical encounters (Registrar, Head and Neck), as supporting and ‘standardizing’ the provision of information across team members (Nurse, Head and Neck Cancer), and as helping patients ‘visualize’ the difference between treatment options: ‘Patients can now see for themselves the actual differences in the choices they have, on a piece of paper. Previously they had to imagine it. Now they can actually see it on a piece of paper, I think that visualization of a concept makes things easier for them to understand’ (Consultant, Breast Cancer). Nevertheless, the utility of Option Grids was not necessarily obvious; the added value had to be experienced: ‘I thought [using the Option Grid] is going to take a lot longer in the clinic. It doesn’t. You are actually doing it at the same time as you are talking, and I don’t think it takes any longer at all. The parents ask you a few more questions, but that’s what they should be doing anyhow. It’s not “them and us” anymore, we’re making a decision between us. It feels like that now.’ (Nurse, Paediatric Tonsillectomy).

As leaders in the teams initiated, and then valued new practices, they became advocates, and then enrolled others, thus demonstrating ‘relational integration.’ In order to implement the interventions, each team member needed to understand their roles and responsibilities in relation to this work. The data revealed a process of trial and error among team members as they sought to ‘work out where to put [the Option Grid]’ (Nurse, Paediatric Tonsillectomy), and to allocate the right tasks, printing, data administration and so on, to the right people. Teams that successfully implemented Option Grids and DQMs were those which developed processes, which were usually led by nurses, to ensure routine use: ‘We’ve now got a pack. That makes a big difference, because before we were scrabbling around…but now we’ve got packs with everything in it, the DQM questionnaire, the Option Grid, it’s all there’ (Nurse, Breast Cancer). One individual put the packs together, others ensured packs were available in consulting rooms. Participants described collective action to define roles in the process. In NPT terms, this is ‘skillset workability.’

Team members wanted to know whether SDM was aligned with existing organizational policies. In NPT terms, they were asking for evidence of ‘contextual integration.’ As one consultant commented, ‘If you haven’t got Board buy-in, if you haven’t got support from that level of management, then everything is an uphill struggle’ (Consultant, Head and Neck). Healthcare professionals reported that visits by senior managers of the Health Board demonstrated organizational support for the program.

### Monitoring benefit, reflecting on value

The data showed that an important element of the program was the effort to measure change. In NPT terms, this equates to ‘reflexive monitoring,’ where the teams wanted to know how new practices affected them and their patients. Providers described the importance of agreeing on how to collect information as well as wanting to view the data to understand differences and trends. We noted how motivated the teams were to receive information about the effectiveness of their new practices, which they then used to reconfigure processes further.

During the program, PDSA cycles were used and patients were asked to complete DQMs (see Table 1). Information from PDSA cycle reports and DQMs were appraised in small group meetings or during team feedback events, held on a six-month basis, and contributed to the implementation process. As one consultant stated, ‘The feedback has really helped us as a team to see [where] we were doing well…and also see where a lot more input was needed’ (Consultant, Breast Cancer). Providers felt the feedback sessions increased awareness of how ‘change [was] achieved in practice’ (Consultant, Breast Cancer). This monitoring brought teams together around a common goal of improving practice, yet there were different motivations for the use of the data. Senior clinicians wanted the data for external use, such as to defend relatively high mastectomy rates: ‘When questions about the number of mastectomies versus the number of wide local excision are asked, we can show that our patients have made a quality decision as opposed to following somebody’s preferences or just being told what to do’ (Nurse, Breast Cancer). Nevertheless, there was initial skepticism about the data collection proposal; ‘I think at first we thought: is this going to be a paper exercise?’ (Nurse, Breast Cancer). However, after using DQMs, the breast care nurses came to value both the aggregate data as well as immediate data from individual patients: ‘Ah right, she still hasn’t understood that point…I’ll just make sure they understand exactly what’s going to happen.’ As the nurses realized the real time value of these data, they took on responsibility for collecting DQMs regularly.

In NPT terms, the effort to measure and discuss the results is evidence of ‘reflexive monitoring.’ Data collection was far more likely when those responsible for data collection perceived the process to be relevant and beneficial. Teams that routinely monitored new SDM practices could demonstrate impact, which increased motivation for sustained implementation.

## Conclusions

### Principal findings

This study showed that the implementation of SDM is far more complex than the delivery of patient decision support, which is how many implementation efforts have been conceptualized so far [1]. We found that healthcare providers had divergent attitudes toward the concept of sharing decisions with patients, and that this was accentuated by efforts made to implement SDM. Normalizing shared decision making requires intensive work to ensure that teams have a shared understanding of the purpose of involving patients in decisions, and undergo the attitudinal shifts that many health professionals feel are required when comprehension goes beyond initial interpretations. Divergent views on the value of engaging patients in decisions remain a significant barrier to implementation.

At the outset, many health professionals reported that they were ‘already doing SDM’ and demonstrated limited motivation to change practice. Providing SDM workshops and working collaboratively to develop decision support tools helped individuals to understand that SDM goes well beyond a general involvement of patients, and requires deliberate efforts to inform patients about reasonable treatment options and ascertain preferences. However, increased understanding does not necessarily lead to wide agreement about the desirability of SDM. This core issue refers to ‘coherence,’ a term used by NPT to describe the sense-making work that people do individually and collectively when they are faced with the problem of operationalizing a set of practices. Achieving ‘coherence’ within each of all three specialties proved too difficult. We observed partial implementation, led mainly by nursing colleagues, who were sympathetic to the idea that patients should be offered information and the opportunity to participate meaningfully in making key decisions. While other elements of the NPT were observed – ‘cognitive participation,’ ‘collective action,’ and ‘reflexive monitoring’ ‘coherence’ appeared to be the prerequisite toward successful normalization of SDM.

### Strengths and weaknesses of the study

The strength of this study is the longitudinal nature of the design and the nature of the interview data captured at two critical time points. Interviews were conducted by the program facilitator (AL), who worked with the teams to support their engagement. The interviewer had an in-depth knowledge of the efforts she and others had made to introduce the interventions to the teams (see Table 1), and had good working relationships with all the teams’ members. The interviews were candid; the providers did not feel obligation to the program to report success, as is evident from the transcripts. Two individuals (AR and AL) independently coded the transcripts, and our interpretation of the transcripts was also discussed with members of the clinical teams for respondent validation.

We were able to talk to most team members but not all, and it is likely that providers not interviewed would have been even more critical, given their unwillingness to contribute. We acknowledge that this is a small sample, and that our data may not be generalizable to other clinical teams. We are unsure whether specific attributes of the clinical specialties influenced the applicability of SDM, although we note that the issues identified in this study are consistent with those found in other similar studies (see below).

We are also unsure whether the specific attributes of the patients influenced their capacity to engage in SDM, as previous studies have suggested [23]. The Research Governance Committee decision that the study did not constitute primary research enabled us to try a number of different interventions. However, we were unable to collect data from patients and therefore lack their perspective in this study.

### Comparison to existing literature

The findings from this study deepen our understanding of implementation challenges already published. Frequently cited issues about health professionals’ lack of understanding about how to operationalize SDM [6,9,24-26] combined with evidence that some health professionals view patient involvement in decision making as inappropriate [8,27] and that poor teamwork hinders implementation [26,28], emphasize the need for ‘coherence.’ Health professionals’ lack of trust in the content of decision support tools [6,8,25,29,30] may be related to low participation in the development and use of such tools. Health professionals frequently report competing demands and time pressures as reasons for failing to incorporate SDM interventions into routine practice [6,9,25-27,29-33], yet this study reveals a deeper issue; even if there were time, at least some providers have and maintain significant ambivalence about the proposal that decisions should be ‘shared.’ Conceptualizing SDM as the provision of ‘information’ to patients puts the central idea of empowering patients at risk. Health professionals willingly accept that patients should be informed: it is much more challenging for them to accept that patient preferences should play a part in how decisions are made [34].

### Implications

Provider ambivalence about SDM, among some team members, impairs ‘coherence’ – an agreed view about ‘the work’ to be done. When coupled with patients’ diffidence (also for varied reasons) about asking questions and disagreeing with providers [23,35], it is no surprise that progress on implementing SDM is slow. Achieving agreement among all team members in how to answer the seemingly innocent question of ‘what is the work?’ is a vital step in the process. It is also essential to evaluate the implementation of SDM from the patient perspective. How to achieve ‘coherence’ in practice is the next logical research question.

## Competing interests

The authors declare that they have no competing interests.

## Authors’ contributions

AL participated in the conception and design of the study, collected and analyzed the data, and drafted the manuscript. NJW participated in the conception and design of the study, managed the coordination and delivery of the MAGIC program in Cardiff, and helped to critically revise the manuscript. AR participated in the conception and design of the study and helped with analysis and interpretation of the data. AE participated in the conception and design of the study and helped to critically revise the manuscript. GE conceived the study, participated in its design and coordination, and helped to draft and revise the manuscript. All authors read and approved the final manuscript.
